# A microbiota-epigenetic circuit controls systematic circadian programs in the gut epithelium

**DOI:** 10.3389/fsysb.2023.1175306

**Published:** 2023-08-08

**Authors:** Junjie Ma, Jianglin Zhang, Zheng Kuang

**Affiliations:** Department of Biological Sciences, Carnegie Mellon University, Pittsburgh, PA, United States

**Keywords:** circadian rhythm, microbiome, intestinal epithelial cells, HDAC3, histone acetylation, metabolism and immunity, cell proliferation

## Abstract

The intestinal microbiota is an important factor that regulates mammalian circadian rhythms and health. We previously reported that the microbiota synchronizes lipid uptake and metabolism in the intestinal epithelium through histone deacetylase 3 (HDAC3). However, the breadth and significance of microbiota-circadian crosstalk in the intestine are not well understood. Here, we show that the gut microbiota programs the rhythmic expression of a broad range of biological processes, and temporally orchestrates epithelial functions and physiology in accordance with the rhythmic gut environment. Protein synthesis, cell proliferation, and metabolic and immune activities are differentially expressed in the daytime and nighttime respectively, indicating a daily alternation of “working” and “recharging” themes in the gut. The rhythms of gene expression are dampened or altered in germ-free mice, suggesting that the microbiota helps to structure the timing of host gene expression. Further analysis showed that HDAC3 drives a vast majority of these microbiota-dependent circadian programs, likely through rhythmic deacetylation of histones. Motif enrichment analysis revealed that HDAC3 could differentially control distinct rhythmic pathways, most likely by recruiting different transcription factors. These findings provide a systematic view of how the commensal microbiota exploits an epigenetic factor to program just-in-time functions in the intestinal epithelium and maintain host homeostasis.

## 1 Introduction

Physiological processes are influenced by day-night occurrences and regulated cyclically by the circadian clock. The circadian clock drives a rhythmic expression of genes and is coupled to the day-night cycle. In mammals, the circadian rhythm is dictated by the master pacemaker in the suprachiasmatic nucleus (SCN) of the hypothalamus (Weaver, 1998). The molecular circadian machinery is often described as a transcription-translation loop (Mohawk et al., 2012). CLOCK and BMAL1 are transcription factors that bind to *Cry* and *Per* genes to promote their expression. Meanwhile, CRY and PER proteins inhibit the CLOCK-BMAL1 heterodimer, which results in the subsequent decrease in the expression of *Cry* and *Per* genes. This feedback loop helps to orchestrate the levels of their target proteins in a day-night manner.

The central clock in SCN entrains peripheral clocks in a hierarchical manner. In this way, the day-night light cycle synchronizes peripheral clocks which control a variety of tissue-specific processes. Among them, the circadian rhythms in the gastrointestinal (GI) tract are essential for nutrient uptake and immune defense functions. In particular, the GI tract experiences an alternation of food intake and fasting as a consequence of the 24-h day/night cycle. The intestinal epithelium is the first layer in the gut to digest food, absorb nutrients and defend against potential pathogen invasion. Importantly, circadian rhythms of gene expression in the intestinal epithelium are controlled by both the core circadian clock and environmental factors, such as feeding schedule and diet composition (Hoogerwerf et al., 2007).

The composition and function of the gut microbiota are entrained with the diurnal oscillation, which is controlled by the rhythmicity of food intake and nutritional content. (Thaiss et al., 2014; Zarrinpar et al., 2014; Leone et al., 2015; Liang et al., 2015). The total number of bacteria (Liang et al., 2015; Risely et al., 2021; Thaiss et al., 2016) and the number of bacteria attached to the intestinal epithelium (Brooks et al., 2021; Thaiss et al., 2016) are high during the feeding period. Thaiss et al. reported that 60% of the total microbiota showed relative abundance rhythmicity in mice. In both mice and humans, Firmicutes and Bacteroidetes, as the two most dominant phyla of the gut microbiome exhibit diurnal rhythmicity, which is related to the feeding phase: Firmicutes peaking at the end of the feeding and Bacteroidetes peaking at the fasting stage (Thaiss et al., 2014). Along with the rhythmicity of the community composition of the microbiome, the bacterial functional composition was also found to be rhythmic, as suggested by the rhythmic expression of microbial pathways, including bacterial motility and mucus degradation (Thaiss et al., 2014; Thaiss et al., 2016). Furthermore, fecal contents from subjects with jet lag were transplanted to GF mice to induce obesity and glucose intolerance due to the disruption of the circadian rhythm (Thaiss et al., 2014). In addition, short-chain fatty acids, as common microbial metabolites, have also been reported to have a rhythmic occurrence (Leone et al., 2015; Tahara et al., 2018; Segers et al., 2019; Segers et al., 2020).

Many facets of the host circadian physiology can be impacted by the presence and rhythmicity of the gut microbiome, including host gene expression (Thaiss et al., 2016; Weger et al., 2019; Kuang et al., 2019), serum metabolites (Thaiss et al., 2016; Weger et al., 2019), immune function (Godinho-Silva et al., 2019; Teng et al., 2019; Wang et al., 2019) and lipid absorption (Wang et al., 2017; Kuang et al., 2019). Generating an animal model with an arrhythmic microbiota in the gut is not achievable currently; therefore, pre-existing studies have been done by ablating the gut microbiota through treatment of antibiotics or by using germ-free (GF) mice. Thaiss et al. reported that various clusters of biological pathways lost or gained rhythmic oscillation in the colons of antibiotics treated mice in comparison to conventional (CV) mice (Thaiss et al., 2016). The omics data suggested that the change of rhythmicity in the host is driven by the alteration of the microbiome.

A variety of pathways have been shown to exhibit daily rhythms. The epithelial cells in the intestine undergo a fast self-renewal process, during which the entire epithelium is replaced every 3–5 days through the activity of stem cells (Heath, 1996; Park et al., 2016). There is sufficient evidence indicating that the cell cycle *per se* is governed by circadian rhythms (Yoshida et al., 2015; Stokes et al., 2017). Besides, it is reported that the diurnal fluctuation of colon epithelial cell proliferation was impacted by the feeding schedule (daytime and nighttime restricted feeding) (Yoshida et al., 2015). Immune defense is another key function in the intestinal epithelium to protect the host from microbes and other toxic substances in the gut. For example, antimicrobial peptides are produced by the intestinal epithelium and the abundance shows circadian rhythms, corresponding to the rhythmic attachment of the segmented filamentous bacteria (Brooks et al., 2021). The expression of toll-like receptors was shown to have circadian rhythms and the rhythms were dampened in antibiotics-treated mice (Mukherji et al., 2013). Additionally, IEC major histocompatibility complex (MHC) class II is diurnally regulated by adherent commensals to maintain the microbiome-epithelial-immune homeostasis (Tuganbaev et al., 2020). Therefore, a diverse range of intestinal epithelial activities can be temporally regulated by circadian rhythms.

We previously generated a circadian dataset of transcriptome and histone acetylation in CV and GF mice and demonstrated that the gut microbiota has a pivotal role in maintaining the rhythms of histone acetylation (e.g., H3K9ac, a mark of active promoters) and gene expression in small intestinal epithelial cells (IEC), and the rhythms are dampened in GF mice (Kuang et al., 2019). We further exploited a *Hdac3*-deficient mouse model where *Hdac3* is specifically deleted in IECs (*Hdac3^ΔIEC^*) and showed that HDAC3, a histone deacetylase is a key molecule mediating microbial regulation of intestinal circadian metabolism. We found that the monocolonization of *Bacteroides thetaiotamicron* that belongs to the phylum of Bacteroidetes was able to activate histone deacetylase 3 (HDAC3) at the expression and protein levels, which could potentially promote the rhythms in GF mice (Kuang et al., 2019). However, several important questions are still not well understood. How does the intestinal epithelium temporally coordinate functionally divergent processes in the intestine, such as cell proliferation, nutrient absorption and immune defense? How do the commensal microbiota and HDAC3 help program the rhythms of these epithelial activities?

To understand these questions, we performed a systematic analysis of the time-course RNA-seq and ChIP-seq data to investigate microbiota-driven rhythms in gene expression. We identified a distinctive rhythmicity feature in CV mice, as exhibited by successive expression of epithelial pathways across the 24-h cycle. The amplitudes of rhythmicity are relatively higher in CV mice compared to the rhythmic transcriptome in GF mice. Furthermore, by analyzing the RNA-seq and ChIP-seq data of H3K9ac in *Hdac3^ΔIEC^* mice, we found that HDAC3 programs rhythmic transcription and epithelial functions in IECs in a gut microbiota-dependent manner, majorly by rhythmic histone deacetylation and recruitment of other transcription factors. Together, our work reveals new facets of intestinal epithelial diurnally regulated by the microbiota and HDAC3, indicating a microbe-epigenetic circuit orchestrating host circadian rhythms and maintaining intestinal epithelial homeostasis.

## 2 Materials and methods

### 2.1 RNA-seq data analysis

RNA-seq data (GEO accession ID: GSE134303) was downloaded from GEO and analyzed as previously described (Kuang et al., 2019). *Hdac3*^*fl/fl*^ (Montgomery, et al., 2008) and *Hdac3^ΔIEC^* mice were housed in the SPF barrier at the University of Texas Southwestern Medical Center. *Hdac3*^*fl/fl*^ mice (Montgomery, et al., 2008) were used to create intestinal epithelial cell (IEC)-specific *Hdac3* knockout mice (*Hdac3^ΔIEC^*) by breeding with a mouse expressing Cre recombinase under the control of the IEC-specific Villin promoter (Madison, et al., 2002). Germ-free (GF) C57BL/6 mice were maintained in the gnotobiotic mouse facility at the University of Texas Southwestern Medical Center as described (Cash, et al., 2006). All mice were housed under a 12-h light/12-h dark cycle. Mice were fed *ad libitum*.

Sequence data was mapped against the mm10 genome using TopHat (Trapnell et al., 2012) and FPKMs were generated using Cuffdiff (Trapnell et al., 2012) with default parameters. The Elbow method of K-means clustering was used to determine the optimal number (K value) of clusters in the data sets based on calculating the Within-Cluster-Sum of Squared Errors (WSS) for different values of K. Gene expression abundance was then visualized with a heatmap using the “heatmap.2” function in R.

### 2.2 ChIP-seq data analysis

H3K9ac ChIP-seq data (GEO accession ID: GSE134303) was downloaded from GEO and analyzed as previously described (Kuang et al., 2019). Sequence data was mapped against the mm10 genome using Bowtie2 (Langmead and Salzberg, 2012), signals were normalized by the total numbers of aligned reads and visualized using Cisgenome Browser (Ji et al., 2008). The promoter region was defined as −1,500 base pair (bp) to 500 bp relative to the transcription start site (TSS) for each gene and histone acetylation signals were calculated in R using the “countOverlaps” function as previously described (Kuang et al., 2014; Kuang et al., 2017). Relative amplitudes were calculated as (max_H3K9ac - min_H3K9ac)/max_H3K9ac.

### 2.3 JTK_cycle analysis

JTK_CYCLE is a non-parametric rhythm detection method (Hughes et al., 2010) that is widely used in the field of circadian biology to detect rhythmic components in a large-scale dataset of transcripts, proteins, or metabolites and estimate the optimal phase and amplitude. In this study, the analysis was performed by incorporating a window of 18–30 h for the determination of circadian periodicity. Bonferroni-adjusted *p* values <0.05 were considered significant. The Benjamini–Hochberg procedure was used to control the false discovery rate (FDR). JTK_cycle results are provided in Supplementary Table S1. Relative amplitudes were calculated by dividing the amplitudes output by JTK_cycle to the max expression level.

### 2.4 Gene Ontology enrichment analysis

Gene Ontology (GO) analysis was performed using the online tool DAVID Bioinformatics Resources (https://david.ncifcrf.gov/) with the gene list obtained from the previous steps.

With the abundance of significantly enriched GO terms output from DAVID, Rrvgo package (Sayols, 2020) was used to help simplify the redundancy of GO sets by grouping the terms based on the semantic similarity. Treemap charts were used to interpret the summarized GO enrichment results with an area-filling visualization of hierarchical structure, in which the area was proportional to the FDR.

GO pathway term networks with non-redundant biological information were generated by ClueGO (Bindea et al., 2009) integrated in Cytoscape (Shannon et al., 2003), using user-provided gene lists. ClueGO is using kappa score to define term-term interaction and to associate terms and pathways into functional groups based on shared genes (Bindea et al., 2009). Kappa score is the Cohen’s kappa coefficient, which measures how much two observers agree with each other when they are rating categorical items (McHugh, 2012).

$$\text{κ}=\frac{\text{Pr}(a)-\text{Pr}\;(e)}{1-\text{Pr}\;(e)}$$

Pr(a) = the actual observed agreement.Pr(e) = the hypothetical probability of chance agreement.The kappa score was set to 0.4 (default) to link interacting terms in the network and only the pathway terms with a significance level≤0.05 were shown. The final pathway networks were visualized with the predefined significance color code.

### 2.5 Transcription factor motif enrichment analyses

Motif analyses were performed as previously described (Kuang et al., 2017). CisGenome was used to map transcription factor (TF) binding motifs to the mm9 genome (528 motifs from TRANSFAC (Wingender et al., 2001). Motif sites with likelihood ratio >500 (i.e., the default cutoff value of CisGenome) were reported, and their locations were used for subsequent analyses. Motif sites that are located at −900 bp to 100 bp of a given gene’s TSS were identified by the “countOverlap” function in R. To test whether a motif is enriched in a given gene list, Fisher’s exact tests were conducted using the following four numbers: the number of genes containing the TF motif from the list (x1), the number of genes not containing the TF motif from the list (x2), the number of genes containing the TF motif from the genome (z1), the number of genes not containing the TF motif from the genome (z2). *p*-values from the tests were adjusted by the “p.adjust” function to calculate the false discovery rate (FDR) with the “fdr” method to account for multiple comparisons.

## 3 Results

### 3.1 A myriad of pathways are temporally orchestrated in the intestinal epithelium of CV mice

**FIGURE 1:**
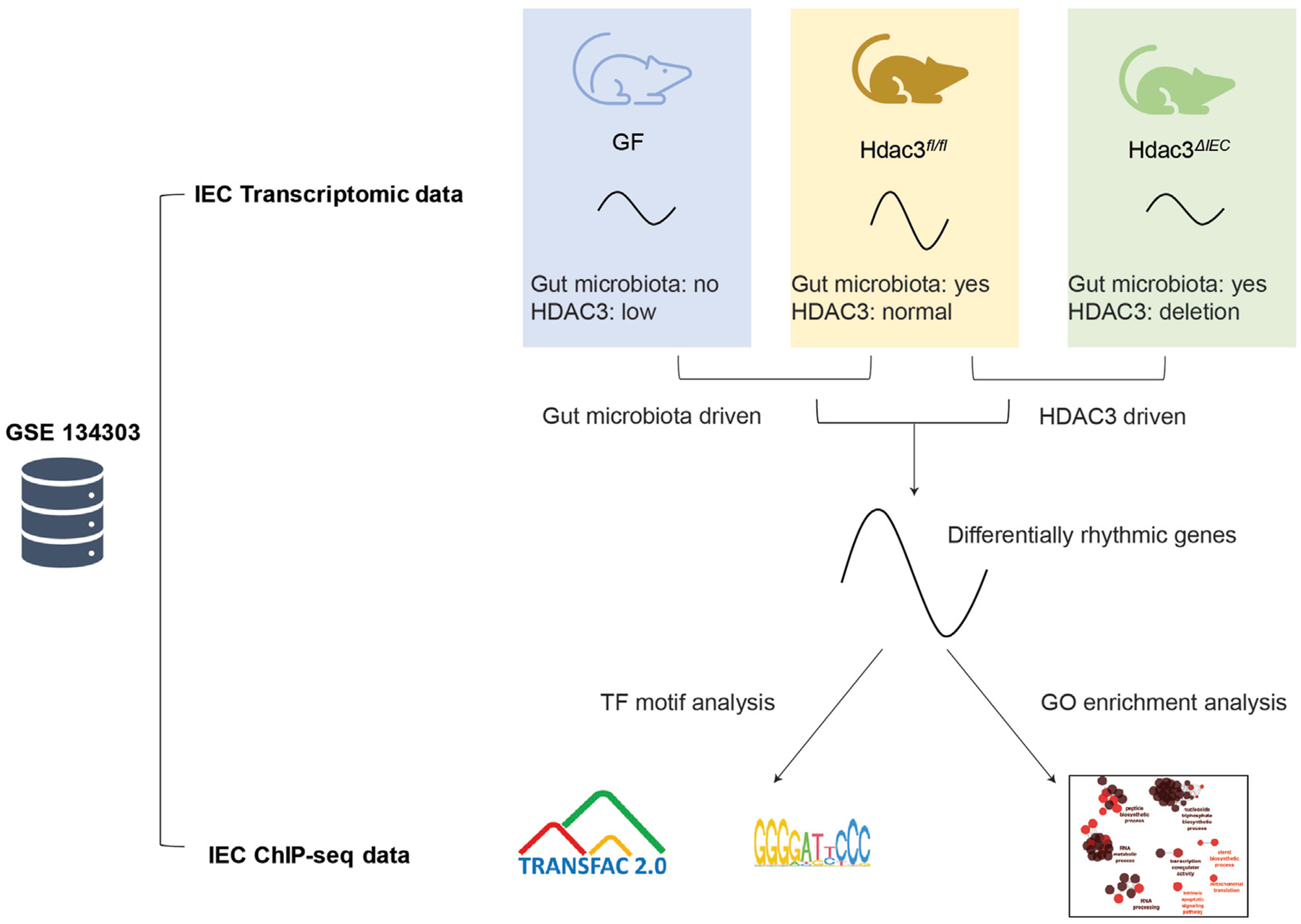
Schematic illustration of the systematic rhythmicity analysis on transcriptomic and ChIP-seq data from *Hdac3*^*fl*/*fl*^, *Hdac3*^*ΔIEC*^ and GF mice.

**FIGURE 2:**
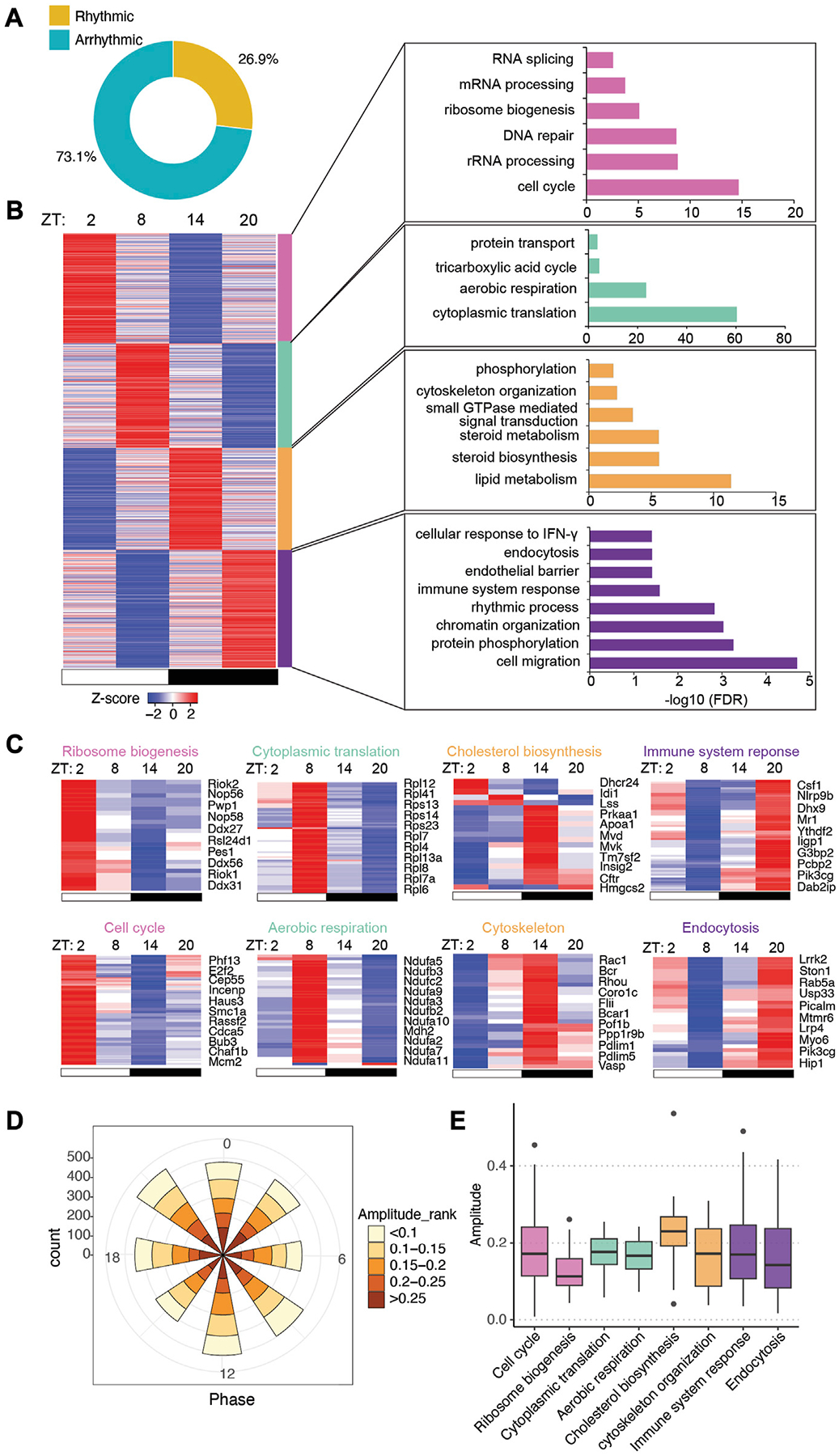
Intestinal epithelial pathways undergo diurnal fluctuations in CV mice. **(A)** Pie-chart indicating the number of transcripts identified as rhythmic (yellow) (Bonferroni adj. *p*-value ≤0.05) by JTK_cycle and arrhythmic (teal) (Bonferroni adj. *p*-value >0.05). **(B)** Heatmap representation (left) of cycling transcripts in 4 clusters and GO analysis (right) within each cluster (cluster 1: magenta-pink, cluster 2: green, cluster 3: orange, cluster 4: purple). **(C)** Heatmap depicting representative GO pathways within each cluster, annotated by the color for each cluster. **(D)** Polar chart displays peak phase distribution of the amplitude of rhythmic genes in CV mice. The amplitude is the relative amplitude (see Materials and methods). The relative amplitude values range from <0.1 to >0.25, which were categorized into 5 ranks: <0.1, 0.1–0.15, 0.15–0.2, 0.2–0.25, >0.25 and indicated by the color scale from light to dark. **(E)** Box plot of amplitudes of the oscillating pathways from **(C)**.

To determine how the gut microbiota regulates circadian rhythms in the intestinal epithelium (Figure 1), we previously performed time-course RNA-seq across a day/night cycle in IECs from GF and CV wild-type (WT) mice (Kuang et al., 2019). We found that the gut microbiota was able to drive the oscillation of gene expression in nutrient transport and lipid metabolism in the small intestine. Here, we exploited the JTK_cycle algorithm and GO enrichment to present a systematic view of the circadian gene expression program in mouse IECs (Figure 1). A total of 6,270 out of 23,285 transcripts significantly oscillate on the level of expression abundance (Figure 2A, adjusted *p* < 0.05). Rhythmic transcripts have higher expression abundance compared with arrhythmic transcripts (Supplementary Figure S1A), and were enriched in a wide range of molecular, cellular and physiological processes, such as cytoplasmic translation, ATP synthesis, protein transport and cell division (Supplementary Figure S1C, D). The size of the space area on the treemap chart exemplifies the significance of the enrichment result (Supplementary Figure S1E).

To understand the significance of gene expression timing in host-environmental interactions, we clustered rhythmic transcripts into four groups by K-means (Supplementary Figure S1B) and visualized the expression patterns by a heatmap (Figure 2B). GO analysis showed that biological processes were temporally orchestrated at different zeitgeber times (ZT) (where ZT0 is light on and ZT12 is light off) across the 24-h light-dark cycle (Figures 2B, C). Starting from ZT14, around the beginning of the night, metabolic pathways such as lipid metabolism and cholesterol biosynthesis are highly upregulated, presumably in correspondence to the increment of feeding activities in mice. Immune response pathways are activated near the end of the night (i.e., ZT20), which may help the host address the expansion of gut microbes after food intake. Pathways in cytoskeleton organization and endocytosis are also upregulated during the nighttime. At ZT2 and ZT8, during the daytime when mice are less active, transcripts related to cell cycle, aerobic respiration, ribosome biogenesis and cytoplasmic translation are highly expressed, suggesting that the intestinal epithelium could be recharging and replenishing the population of epithelial cells.

The polar chart of all rhythmic transcripts showed evenly distributed amplitudes of expression at each phase across the 24 h (Figure 2D). Notably, individual biological pathways have different oscillatory amplitudes of expression abundance, with cholesterol biosynthesis having the highest amplitude among the presented pathways (Figure 2E). Together, our RNA-seq analysis revealed distinct circadian gene expression programs of different functional pathways in CV intestinal epithelium, and indicated a temporal alternation of “working” (e.g., nutrient uptake and metabolism, immune functions) and “recharging” (e.g., protein synthesis and cell cycle) in response to the fluctuating levels of nutrients and microbes in the gut.

### 3.2 The intestinal transcriptome exhibits an altered circadian pattern in GF mice

**FIGURE 3:**
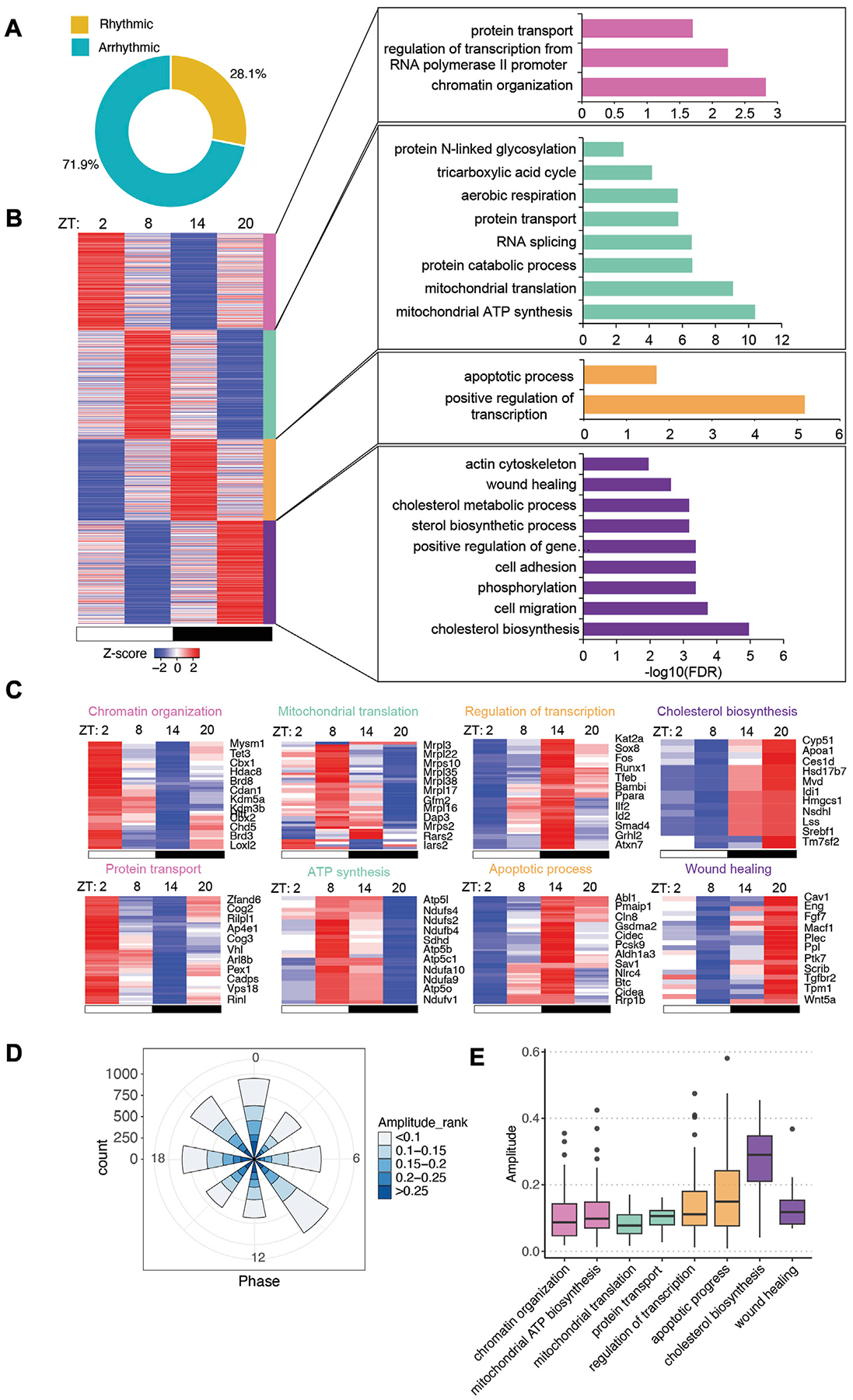
Intestinal epithelial pathways undergo diurnal fluctuations in GF mice with lower amplitude. **(A)** Pie-chart indicating the number of transcripts identified as rhythmic (yellow) (Bonferroni adj. *p*-value ≤0.05) by JTK_cycle and arrhythmic (teal) (Bonferroni adj. *p*-value >0.05). **(B)** Heatmap representation (left) of cycling transcripts in 4 clusters and GO analysis (right) within each cluster (cluster 1: magenta-pink, cluster 2: green, cluster 3: orange, cluster 4: purple). **(C)** Heatmap depicting representative GO pathways within each cluster, annotated by the color for each cluster. **(D)** Polar chart displays peak phase distribution of the amplitude of rhythmic genes in GF mice. The amplitude is the relative amplitude (see Materials and methods). The relative amplitude values range from <0.1 to >0.25, which were categorized into 5 ranks: <0.1, 0.1–0.15, 0.15–0.2, 0.2–0.25, >0.25 and indicated by the color scale from light to dark. **(E)** Box plot of amplitudes of the oscillating pathways from **(C)**.

The gut microbiota has been shown to impact host circadian rhythms significantly (Thaiss et al., 2016; Weger et al., 2019; Kuang et al., 2019). To gain more insight into how the microbiota helps to tune the rhythmicity of host gene expression, we examined the circadian rhythm of transcriptome in the small intestinal epithelial cells of GF mice. Using the JTK_cycle algorithm, we found that 6,543 of the 23,285 genes were significantly oscillating (Figure 3A, adjusted *p* < 0.05). We exploited the same pipeline to analyze the rhythmic genes in GF mice and found that rhythmic transcripts had higher expression than arrhythmic transcripts (Supplementary Figure S2A).

With a similar K-means clustering strategy (Supplementary Figure S2B), we grouped GF rhythmic transcripts in four clusters. Unlike the CV transcriptome, a majority of functional pathways were enriched at ZT8 and ZT20 while fewer pathways were overrepresented in clusters 1 and 3 (Figure 3B). Several pathways are rhythmic in both CV and GF mice, including chromatin organization, aerobic respiration, RNA splicing, actin cytoskeleton organization, cholesterol metabolism and cell migration (Figures 3B,C). However, many of them exhibited shifted circadian phases compared to CV mice, such as sterol biosynthesis and RNA splicing. A few pathways were uniquely enriched in GF rhythmic transcripts including apoptotic process and wound healing, which peaked at nighttime. Intriguingly, low-amplitude rhythmic transcripts were enriched in GF IECs (Figure 3E). Among the presented biological pathways, cholesterol biosynthesis had the highest amplitude, similar to CV mice. Additionally, GO analysis of all the rhythmic genes showed that positive regulation of transcription and protein transport were the two major enriched pathways (Supplementary Figure S1C–E). Together, these data revealed a different landscape of circadian gene expression in GF mouse intestinal epithelium.

### 3.3 The circadian rhythm of the IEC transcriptome is reprogrammed in GF mice

**FIGURE 4:**
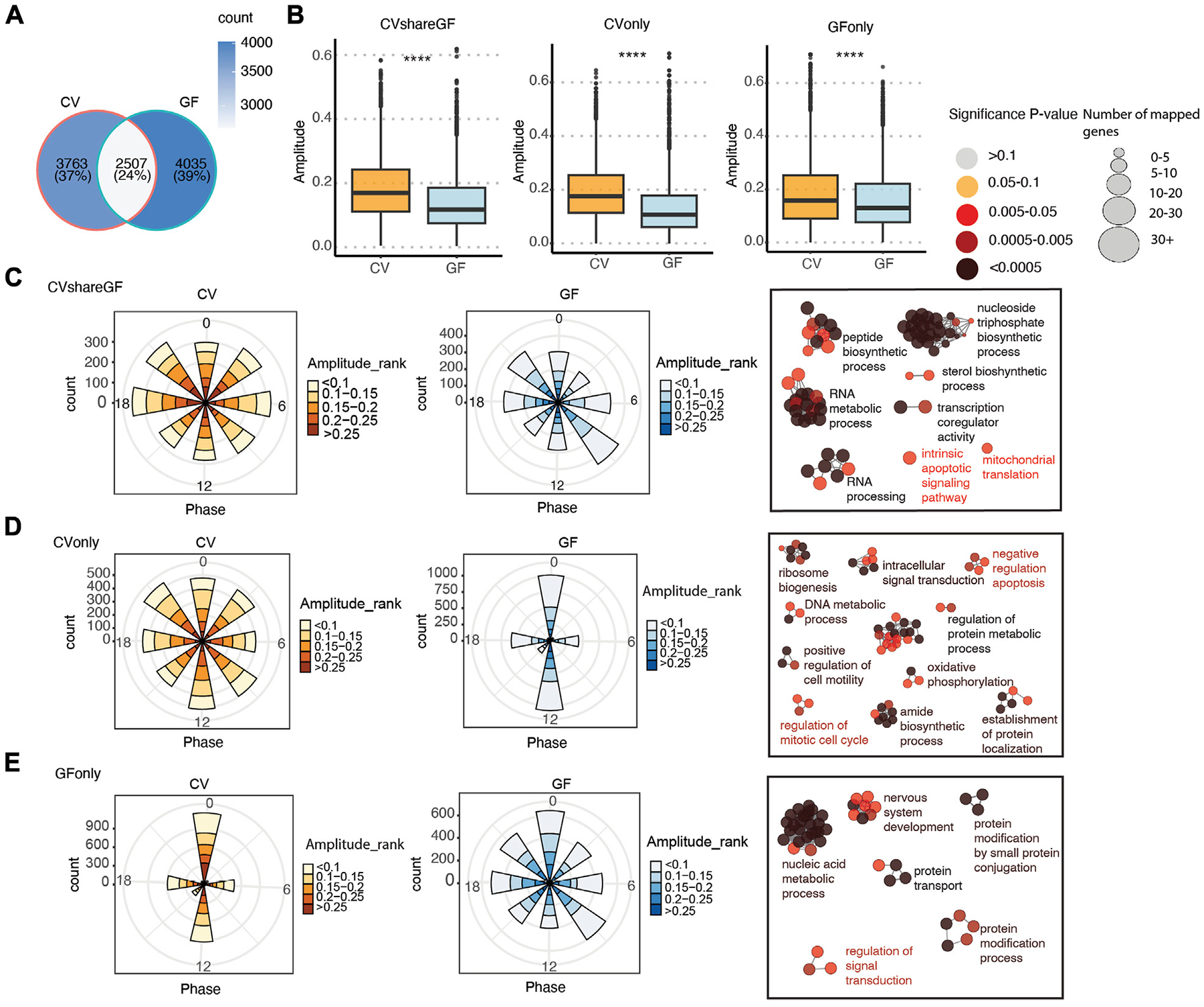
The circadian rhythm of gene expression in IECs is programed differentially in CV and GF mice. **(A)** Venn diagram of shared and unique oscillatory transcripts in IECs of CV and GF mice. **(B)** Comparisons of the amplitude between CV and GF mouse in transcripts of the “CVshareGF”, “CVonly” and “GFonly” groups respectively. ****p < 0.0001 shown by Student’s t-test. **(C–E)** Polar chart displays peak phase distribution of the amplitude of rhythmic genes in “CVshareGF” **(C)**, “CVonly” **(D)**, “GFonly” **(E)**, and functionally organized GO term networks on the right side. The amplitude is the relative amplitude (see Materials and methods). The relative amplitude values range from <0.1 to >0.25, which were categorized into 5 ranks: <0.1, 0.1–0.15, 0.15–0.2, 0.2–0.25, >0.25 and indicated by the color scale from light to dark. The size of node in GO term networks **(C–E)** means the number of mapped genes, which is proportional to the size of the nodes.

Given the role of the microbiota in modulating host circadian rhythms, we next systematically compared the circadian gene expression pattern and rhythmic biological processes between CV and GF mice. About 40% of the rhythmic genes in CV mice remained cycling in GF mice (labeled as “CVshareGF”) (Figure 4A), and the other 60% that only oscillated significantly in CV mice were denoted as “CVonly”. The genes with rhythmic expression only in GF mice were denoted as “GFonly”. All the three groups appeared to have a relatively higher amplitude of circadian expression in CV mice, with the “CVonly” group showing the highest difference (Figures 4B–D). It suggests that the gut microbiota may be crucial in maintaining the high-amplitude circadian rhythmicity in IECs. Interestingly, the phases of arrhythmic transcripts in both the “CVonly” and “GFonly” group were presented as singularized phase distribution at ZT0 and ZT12 (Figure 3D middle, 3E left). Conversely, the amplitude distribution of the rhythmic genes was more evenly allocated at each phase across the 24-h cycle (Figures 4C–E). Within each phase, GF mice have more low-amplitude transcripts compared with CV mice (Figures 4C–E). These results corroborated the significance of the gut microbiota in shaping host circadian gene expression via enhancing rhythmic amplitudes.

We further investigated the functions of genes within each group via ClueGO-based GO enrichment analysis (Bindea et al., 2009). Biological processes, including RNA metabolism, nucleoside triphosphate biosynthesis were enriched in the “CVshareGF” transcripts (Figure 4C). Transcripts associated with ribosome biogenesis, amide biosynthesis, oxidative phosphorylation, and regulation of protein metabolism and cell cycle were highly enriched in the “CVonly” cluster (Figure 4D). This result suggested that the gut microbiota plays a substantial role in orchestrating the circadian rhythm of energy metabolism, protein synthesis and cell proliferation. Finally, the “GFonly” group was enriched with transcripts related to nucleic acid metabolism, protein modification and protein transport pathways (Figure 4E). Altogether, these results suggest that the gut microbiota helps to synchronize a variety of essential cellular and physiological processes that demand energy and biomolecules.

**FIGURE 5:**
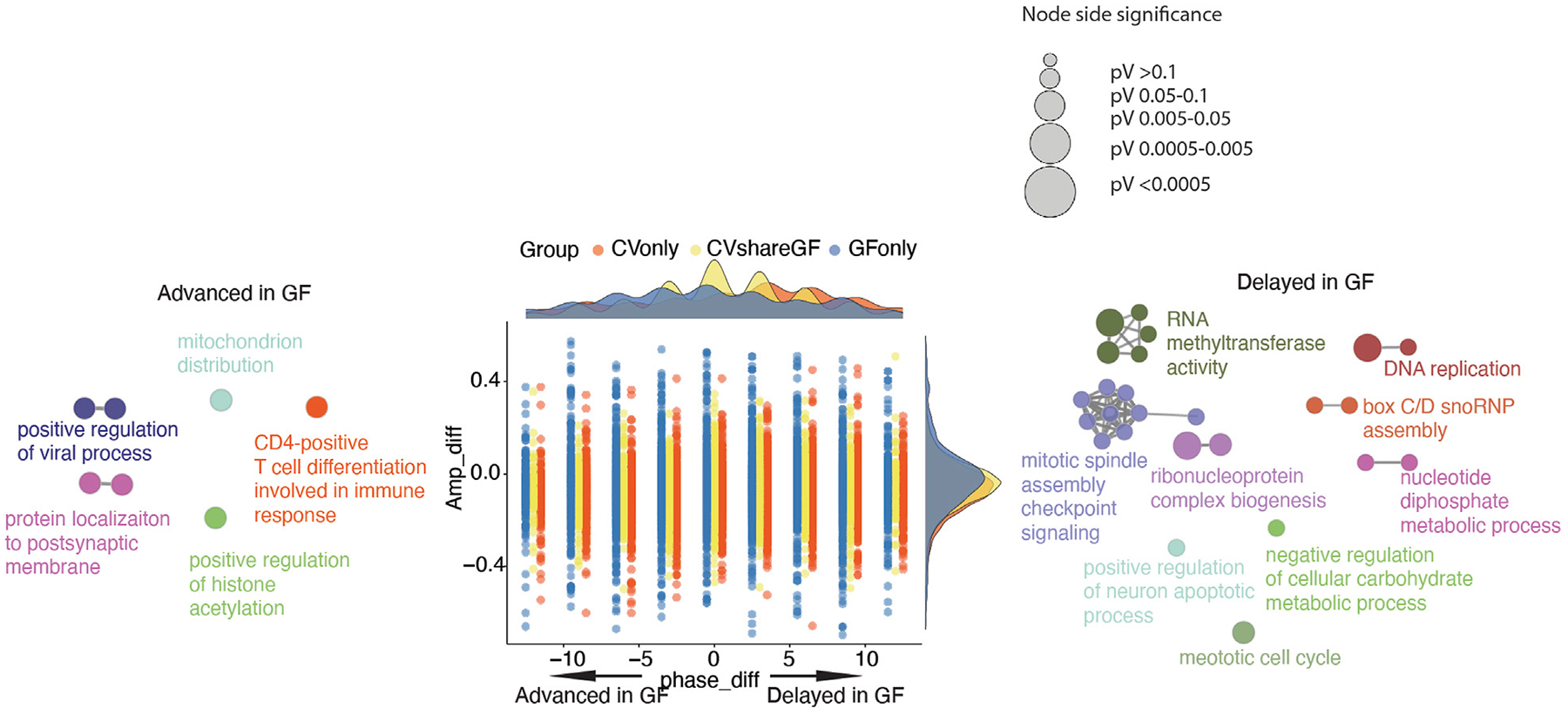
Phase and amplitude analysis of rhythmic genes in CV and GF mice. Phase_diff is defined as phase1-phase2; Amp_diff is defined as Amplitude1-Amplitude2. One and 2 denotes the same gene of GF and CV mice respectively. Dots (genes) located on the left side (<0) means the circadian is advanced in GF mice, while dots on the right side (>0) means the circadian is delayed in GF mice. Density plots are used to show the distributions of either amp_diff (right) or phase_diff (top) for each comparison (CVshareGF: yellow, CVonly: orange, GFonly: grey). GO term network of genes in “CVshareGF” group with phase shifted left, and GO term network of genes in “CVshareGF” group with phase shifted right and the size of node is proportional to the significance level.

Our previous analysis suggested that the microbiota may also shift the circadian phases of gene expression in IECs (Figure 3). Therefore, we investigated the differences of phases and amplitudes (GF vs. CV) within each group of rhythmic genes (i.e., CVshareGF, CVonly and GFonly). As shown in Figure 5, the oscillating genes in the “CVonly” group demonstrated a “right-shift” in GF mice, suggesting a delayed expression timing due to the lack of the gut microbiota. We next investigated the transcripts in the “CVshareGF” group with the circadian phase shifted to both sides. Compared with the circadian rhythm in CV mice, the transcripts having the peak expression occurred earlier (left-shifted) in GF mice were significantly enriched with a few pathways, including immune response and positive-regulation of viral processes (Figure 5). The other cluster of transcripts with a phase-delayed rhythmicity in GF mice were mainly found to regulate the cell cycle. Thus, our results suggest that the gut microbiota regulates both the amplitudes and the timing of rhythmic pathways in mouse intestinal epithelium.

### 3.4 Gut microbiota regulates epithelial circadian rhythms through HDAC3

**FIGURE 6:**
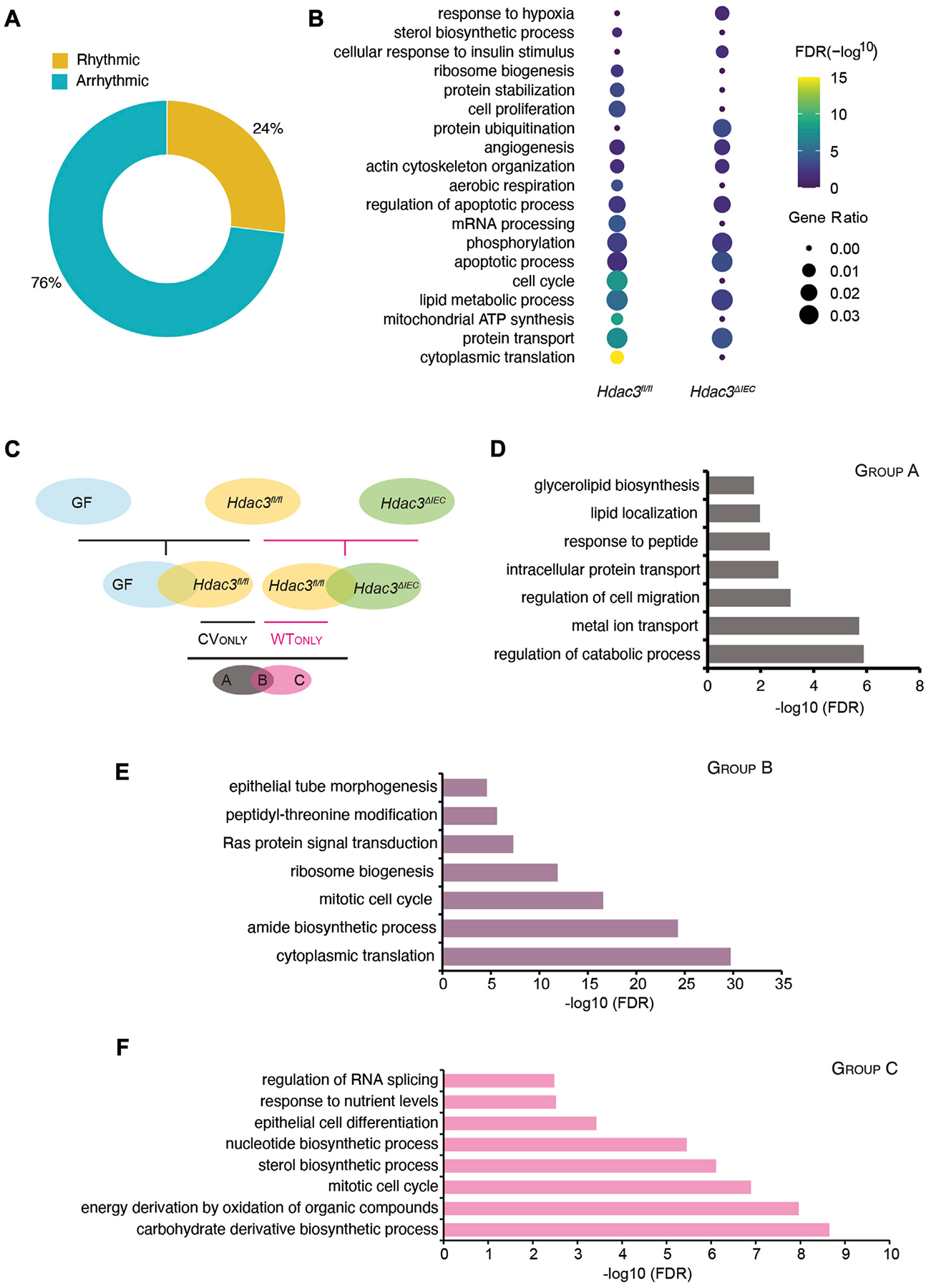
HDAC3 orchestrates the circadian oscillation in the IEC transcriptome together with the gut microbiota. **(A)** Pie-chart indicating the number of transcripts identified as rhythmic (yellow) (Bonferroni adj. *p*-value ≤0.05) by JTK_cycle and arrhythmic (teal) (Bonferroni adj. *p*-value >0.05) in *Hdac3*^*ΔIEC*^ (KO) mice. **(B)** Heatmap showing significantly enriched biological functions in *Hdac3*^*fl*/*fl*^ (WT) and KO rhythmic genes. The size of the dot indicates the gene ratio which is calculated by dividing the number of genes enriched in the individual term by the total number of the input genes. **(C)** Schematic illustration for generating the three groups of genes regulated by the microbiota or HDAC3 (Group A, Group B and Group C). **(D–F)** GO enrichment analysis in transcripts of Group A **(D)**, Group B **(E)** and Group C **(F)**.

Previously we reported that the microbiota activates HDAC3 in the small intestinal epithelium, which drives genome-wide oscillations of histone acetylation and the rhythms of nutrient uptake and metabolic pathways (Kuang et al., 2019). Here we systematically investigated how it regulates the circadian rhythms of gene expression in the intestinal epithelium. First, we applied the JTK_cycle algorithm and identified 5,584 out of 23,285 transcripts that were significantly rhythmic (Figure 6A) in *Hdac3^ΔIEC^* mice. 2,176 transcripts were cycling in both WT (*Hdac3*^*fl/fl*^) and *Hdac3^ΔIEC^* mice (Supplementary Figure S3A) and were defined as the “WTshareKO” group. Transcripts oscillating only in *Hdac3*^*fl/fl*^ mice had a higher amplitude compared with that of *Hdac3^ΔIEC^* mice, implicating HDAC3 as a co-regulator along with the gut microbiota to program epithelial circadian rhythms (Supplementary Figure S3B). The similar result was observed in Figure 4, where “CVonly” transcripts showed higher amplitudes. Another interesting observation was that more “WTshareKO” transcripts peak at ZT12 and ZT15 in *Hdac3^ΔIEC^* mice compared to the phase distribution in *Hdac3*^*fl/fl*^ mice (Supplementary Figure S3B). Additionally, singularized phase distributions at ZT0 and ZT12 was observed for arrhythmic transcripts in both the “WTonly” and “KOonly” groups (Supplementary Figure S3D, E). These results suggest that HDAC3 regulates both the amplitudes and phases of epithelial circadian programs.

To understand the functional pathways that are regulated by HDAC3, we performed a GO analysis of rhythmic transcripts in *Hdac3*^*fl/fl*^ and *Hdac3^ΔIEC^* mice. We found that several pathways were cycling in *Hdac3*^*fl/fl*^ mice but not in *Hdac3^ΔIEC^* mice based on gene ratio, including cytoplasmic translation, mitochondrial ATP synthesis, cell cycle and mRNA processing (Figure 6B). Additionally, lipid metabolic process and protein transport were less enriched in *Hdac3^ΔIEC^* mice compared to *Hdac3*^*fl/fl*^ mice (FDR). Next, we examined the three groups of genes, “WTshareKO”, “WTonly” and “KOonly”. We found that several pathway clusters were shared between “WTonly” and “CVonly” rhythmic transcripts, including energy production, ribosome biosynthesis, translation, and cell cycle. These results indicate that the rhythmicity of these pathways are very likely regulated by both gut microbiota and HDAC3. Meanwhile, compared with “WTshareKO” and “KOonly”, transcripts in the “WTonly” group present the most abundant functional categories in the pathway network (Supplementary Figure S4C–E). It suggests that HDAC3 is critical for maintaining the fundamental biological functions on the level of GO categories as the deletion of which led to the loss of the exact network structure (Supplementary Figure S4D).

To further investigate the regulatory roles of the gut microbiota and HDAC3 in programming circadian rhythms in IECs, we defined three groups of the rhythmic genes and denoted them as “A”, “B” and “C” (Figure 6C). Rhythmic genes in GF, CV *Hdac3*^*fl/fl*^ and *Hdac3^ΔIEC^* mice were subjected to a two-level comparison funnel, aiming to collect genes whose oscillating expression might be driven by the gut microbiota and HDAC3. The first-level comparison is performed within two pairs of groups, which are GF vs. CV *Hdac3*^*fl/fl*^ and CV *Hdac3*^*fl/fl*^ vs. CV *Hdac3^ΔIEC^*. The result generated two clusters of gene groups that we are interested in and they are “CVonly” (compared with GF) and “WTonly” (compared with *Hdac3^ΔIEC^*). Further, we carried out the next level of comparison between “CVonly” and “WTonly”, resulting in three groups and their expression rhythms could be driven by: A-gut microbiota; B-gut microbiota and HDAC3; C-HDAC3. We then investigated the biological functions of the genes in these groups (Figure 6D). Interestingly, we found that the B group of genes were significantly enriched for the biological functions of ribosome biosynthesis, translation and cell cycle, while the A group of genes were more functionally related to the catabolic process, along with metal ion, protein transport and cell migration (Figure 6E). The C group of genes, whose circadian rhythms were likely driven by HDAC3, were enriched with several interesting metabolic pathways, including sterol biosynthesis, carbohydrate derivative biosynthesis, and energy derivation by oxidation (Figure 6F). The functional pathway analysis suggested that the gut microbiota and HDAC3 may drive and tune the circadian rhythms of preferred biological functions in both collective and independent manners.

**FIGURE 7:**
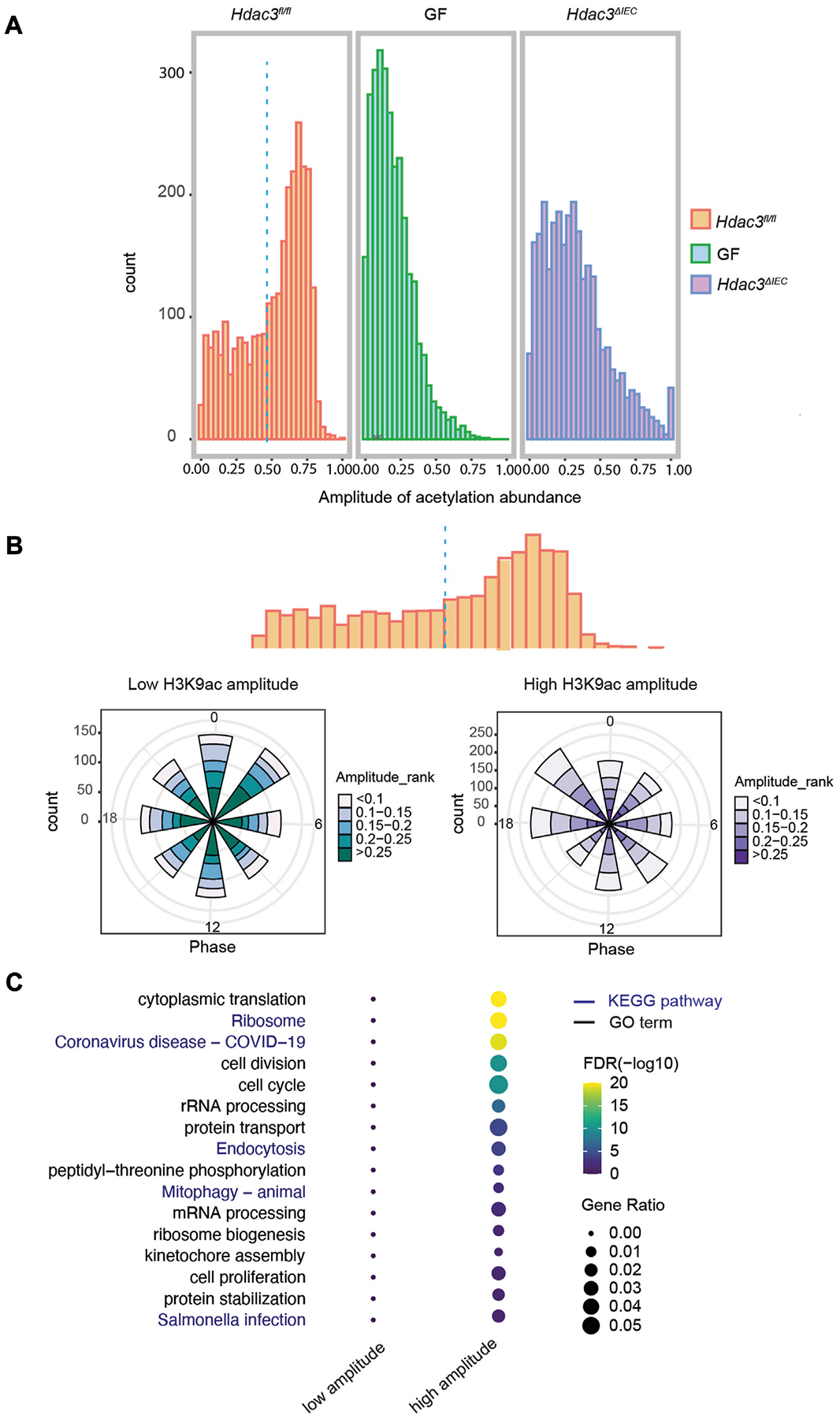
HDAC3-dependent histone acetylation mediates microbial regulation of circadian programs in IECs. **(A)** Histograms of the amplitudes of H3K9ac at group B genes in WT, GF and KO mice. **(B)** Polar histogram plot of the peak phase and amplitude of the rhythmic transcripts with lower (left) and higher (right) histone acetylation amplitude. Lower acetylation amplitude: amplitude <0.5, higher acetylation amplitude: amplitude >0.5. The amplitude is the relative amplitude (see Materials and methods). The relative amplitude values range from <0.1 to >0.25, which were categorized into 5 ranks: <0.1, 0.1–0.15, 0.15–0.2, 0.2–0.25, >0.25 and indicated by the color scale from light to dark. **(C)** Heatmap representation of significantly enriched GO terms and KEGG pathways of the genes with higher or lower acetylation amplitude.

A major mechanism by which HDAC3 regulates gene expression is through histone acetylation. We analyzed the amplitudes of histone acetylation (H3K9ac) at the B group of genes (circadian rhythms driven by both the microbiota and HDAC3), and compared the signals among *Hdac3*^*fl/fl*^, *Hdac3^ΔIEC^* and GF mice (Supplementary Table S2). A majority of the transcripts exhibited high amplitudes of H3K9ac in *Hdac3*^*fl/fl*^ mice, while the amplitudes were much lower in GF and *Hdac3^ΔIEC^* mice (Figure 7A). Interestingly, we observed a similar amplitude distribution in both GF and *Hdac3^ΔIEC^* IECs implying that both the microbiota and HDAC3 may promote circadian gene expression through histone acetylation. We then set the amplitude of 0.466 as a cutoff to divide these transcripts into two clusters based on a statistical analysis of the H3K9ac level in *Hdac3*^*fl/fl*^ mice, aiming to study how histone acetylation regulates rhythmic expression. The polar charts of the two gene clusters indicate that more transcripts from the high acetylation amplitude group (>0.466) had peak expression at ZT18 and ZT21, compared with a phase enrichment at the ZT0 and ZT3 in low-amplitude cluster of genes (Figure 7B). We further performed a GO analysis on these two clusters of genes and observed a striking difference of the enriched biological pathways (Figure 7C). On the dot-heatmap, we combined the significantly enriched biological themes and KEGG pathways (FDR<0.05). A majority of the biological functions were collectively over-represented in the transcripts with higher acetylation amplitude while no pathways were enriched in the low-amplitude cluster (Figure 7C). Altogether, the data indicate that within the B group of genes, whose expression rhythmicity was co-driven by the gut microbiota and HDAC3, histone acetylation rhythms drive the main flow of gene expression oscillation.

### 3.5 Motif enrichment analysis implicates the collective roles of HDAC3 and TFs in circadian regulation

**FIGURE 8:**
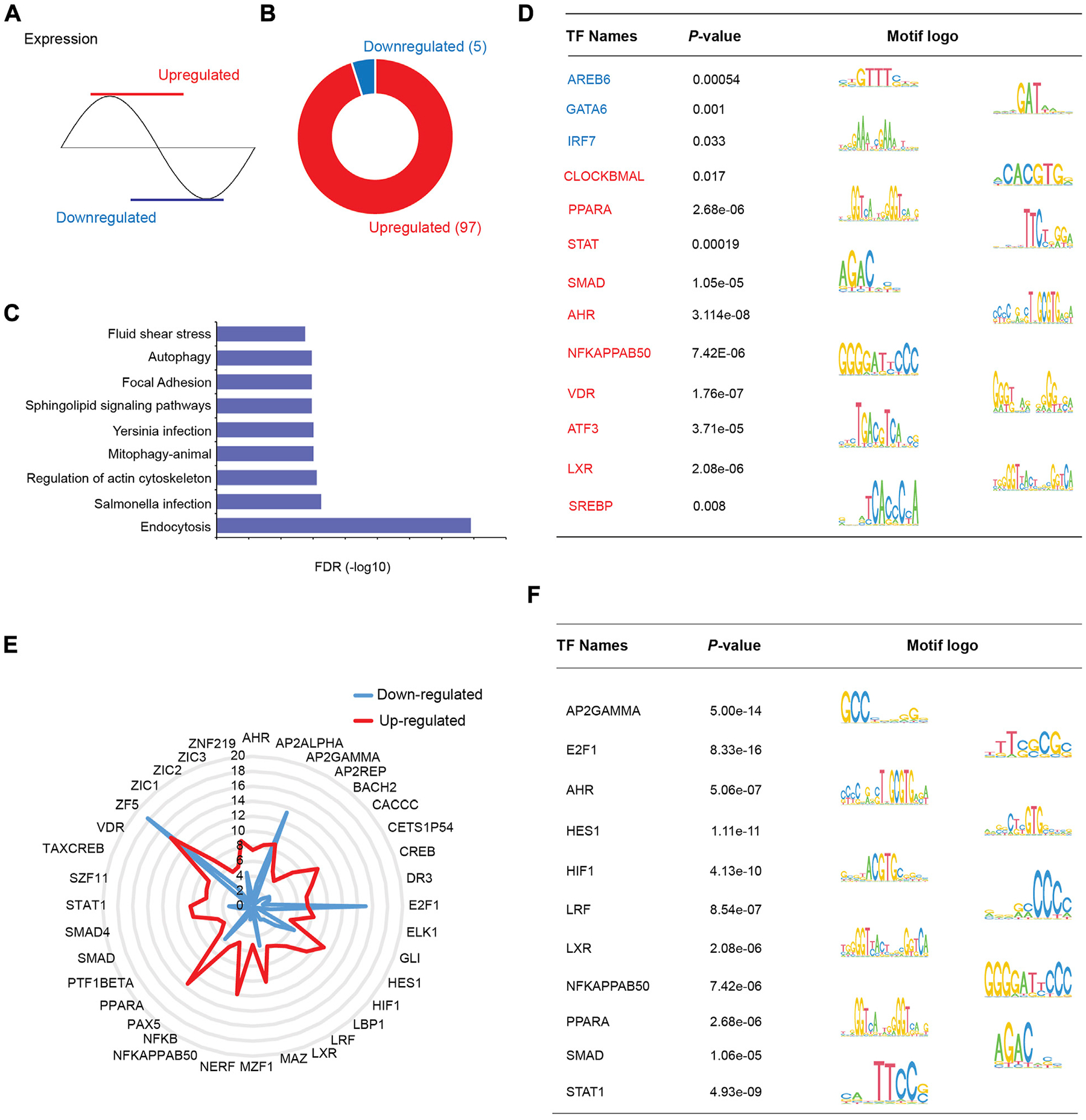
TF motifs are differentially enriched in HDAC3-dependent rhythmic pathways. **(A)** Schematic showing patterns of “upregulated” and “downregulated” transcripts regulated by HDAC3 (upregulated in *Hdac3*^*ΔIEC*^ mice: red, downregulated in *Hdac3*^*ΔIEC*^ mice: blue). **(B)** Pie-chart demonstrating the number of uniquely enriched TF motifs in upregulated and downregulated genes. **(C)** KEGG pathways significantly enriched in upregulated genes (FDR<0.05). **(D)** Summary of individual TF motifs and the corresponding logo view from **(B)**. **(E)** Radar chart showing *p*-values for the most significantly enriched TF motifs in both upregulated and downregulated gene groups. **(F)** Summary of individual TF motifs and the corresponding logo view from **(E)**.

HDAC3 is a transcriptional cofactor and interacts with other TFs to regulate specific downstream pathways. To further investigate the mechanisms by which HDAC3 regulates the circadian rhythms of gene expression, we divided group B rhythmic genes (microbiota and HDAC3-dependent, Figure 6C) into two clusters, “upregulated” (in *Hdac3^ΔIEC^*) and “downregulated” (in *Hdac3^ΔIEC^*) (Figure 8A), and performed TF motif enrichment analysis in each cluster respectively. We found that 97 TF motifs were exclusively enriched in the “upregulated” group while 5 TF motifs were uniquely enriched in the “downregulated” group (Figure 8B). Pathways in endocytosis, infections, actin cytoskeleton and autophagy were enriched in the upregulated transcripts (Figure 8C). Representative enriched TF motifs are shown in Figure 8D, among which SMAD were reported to regulate transcription through histone deacetylase activity (Ross et al., 2006; Tang et al., 2020). The transcription factor CLOCKBMAL is a core circadian clock component known to regulate genome-wide circadian rhythms (Kim et al., 2018). Several lipid metabolism regulators were enriched including PPARA, LXR and SREBP. Immune response regulators were also enriched such as STAT and NF-κB. We consistently observed a majority of TF motifs enriched in the upregulated cluster and these TFs are involved in metabolic and immune pathways and host-microbial interactions (Figure 8E). E2F1, AP2GAMMA and ZF5 were highly enriched in the downregulated gene cluster. Thus, the results suggested that HDAC3 interacts with a variety of TFs to program the circadian rhythms of different pathways in the intestinal epithelium through both canonical and non-canonical mechanisms.

In addition, we examined TF motifs enriched in predefined B group, GFonly group and KOonly genes. The uniquely enriched TF motifs were comparable in each group (Supplementary Figure S4A). One TF motif uniquely enriched in group B genes is PPARG (Supplementary Figure S4B) which is well known to function with circadian proteins (i.e., BMAL1) in an interdependent manner (Chen and Yang. 2014). Besides, PPARG regulates antimicrobial peptide production in a microbiota-dependent manner (Liu et al., 2017), while butyrate generated by the gut microbiota could promote the expression of PPARG concomitantly. This result suggested that the gut microbiota could be a significant environmental component that programs the circadian rhythm of gene expression via PPARG and HDAC3.

## 4 Discussion

**FIGURE 9:**
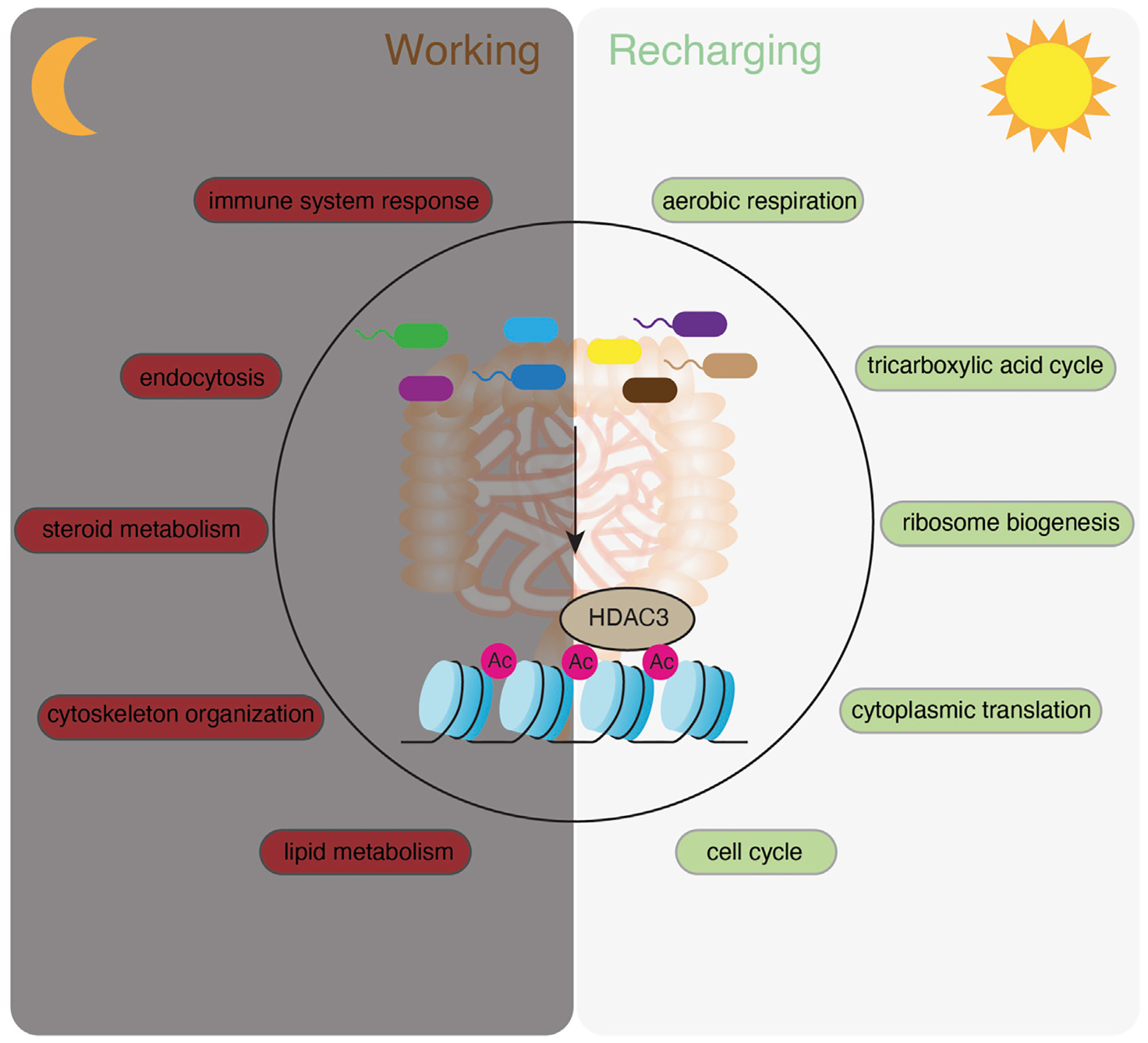
A summary model showing that the microbiota activates HDAC3 and temporally orchestrates “working” and “recharging” pathways across the day/night light cycle to maintain the functionality and homeostasis of the intestinal epithelium.

In this study, we systematically characterized the circadian rhythms of gene expression in mouse IECs and analyzed how the gut microbiota, HDAC3 and transcription factors collectively orchestrate epithelial circadian programs. We found that the gut microbiota drives the circadian rhythms in a variety of molecular, cellular and physiological pathways while maintaining a relatively higher amplitude of gene expression rhythms compared to the GF mice. Interestingly, these rhythmic pathways are synchronized to the daily alternation of active and resting states, exhibited as a “recharging” status during the day and a “working” status at night (Figure 9). Furthermore, we identified TF motifs that are significantly enriched in genes of which the circadian rhythms depend on the microbiota and HDAC3 (Figure 8B), allowing the gut microbiota to program host circadian rhythms via HDAC3 and HDAC3-interacting TFs.

These findings have several important implications. First, our study suggests that the gut microbiota is an essential component in synchronizing host gene expression programs and coordinating epithelial functions in response to the dynamic rhythmic gut environment. While several groups have reported that rhythmic biogeography and metabolome of the intestinal microbiota drives the temporal programming of host transcriptional oscillations (Thaiss et al., 2016; Weger et al., 2019; Kuang et al., 2019), we highlight a microbiota-dependent alternation of “working” and “recharging” programs and provide an explanation for its functional significance (Figure 9). The biological theme of “working” involves extensive host-gut environmental interactions, including nutrient absorption and immune defense which are the two major functions of the intestine. Meanwhile, the intestinal epithelium has to quickly replenish the population and maintain its functionality, which we defined as a “recharging” theme involving mainly intrinsic maintenance and reprogramming processes. We showed that the gut microbiota helps to administer epithelial rhythmicity by arranging the “recharging” theme during the daytime when mice are resting and the “working” theme at night when mice are active and eating. Therefore, with food intake occurring at night (starting from ZT14 in this study), metabolic and relevant cellular pathways are upregulated to increase the capacity for the host to uptake nutrients. It is followed by an increase of immune system response and endocytosis pathways possibly due to the spike of the bacteria abundance in the intestine. Mice are resting in the morning, while intestinal epithelial activities are shifted to the intrinsic “renewal” and “recharging” theme. This is evidenced by upregulation of transcripts related to ATP synthesis, ribosome biogenesis, protein translation and cell cycle. In GF mice, this temporal sequence of gene expression programs is diluted by repetitively enriched functional terms at both day and night, such as the regulation of the transcription, and dampened or shifted functional pathways.

Another interesting observation is that different pathways are globally programmed with varied amplitudes regardless of gut microbiota. For example, the cholesterol biosynthesis pathway has higher amplitude compared with other pathways in both CV and GF mice (Figure 2C; Figure 3C). This could be explained by a recent study in fruit flies (Zhang et al., 2023) suggesting that the higher amplitude prevents random fluctuations in circadian rhythms, which make the organism less sensitive to environmental interruptions. By stabilizing the rhythmicity with a higher amplitude, the organism is not allowed to launch a rapid shift of the circadian rhythm, which could result in difficulties in adaptation to new environments. Another interesting study in humans reported that subjects with lower amplitude of the sleeping circadian rhythm are significantly more resilient and coped better to the sleep loss (Di Milia and Folkard, 2021). It would be interesting to further investigate whether environmental interruptions differentially impact different circadian pathways.

Furthermore, our results support the concept that HDAC3-dependent histone deacetylation is a major motif by which the gut microbiota regulates host circadian rhythms. In this study, we identified a cluster of genes that have strong rhythms of histone acetylation in *Hdac3*^*fl/fl*^ mice and are associated with many essential processes in the intestinal epithelium. However, we did not observe any functional pathways enriched in genes with lower histone acetylation oscillations. This suggests that HDAC3 and the gut microbiota collectively synchronize major biological activities in the epithelium via histone acetylation.

Finally, our study opens new avenues for studying transcriptional and epigenetic regulation of microbial-circadian crosstalks. We showed that several KEGG pathways are detected in the upregulated gene cluster of group B (Microbiota and HDAC3-dependent rhythmic genes), such as endocytosis and enteric infections (Figure 8C). Thereby, studying the causal relationships of these circadian pathways and gut microbiome may shed light on the remaining question about how the gut microbiota persistently impacts host transcriptional rhythmicity. In particular, we identified several significantly enriched TF motifs on the promoter regions of these genes, among which aryl hydrocarbon receptor (AhR) was reported to be promoting fungal invasion via endocytosis (Solis et al., 2017) and the activity of AhR can be induced by many epigenetic factors to impact diverse immunological responses (Wajda et al., 2020). Furthermore, understanding microbial-epigenetic circuits in regulating mammalian circadian rhythms may provide a pinned temporal map for therapeutic approaches to advantage the time of day in terms of restoring the healthy circadian rhythm via alteration of microbiome elements.

## Supplementary Material

Supplementary Table S1

Fig S4

Supplementary Table S2

Fig S3

Fig S2

Fig S1

## Data Availability

Publicly available datasets were analyzed in this study. This data can be found here: https://www.ncbi.nlm.nih.gov/geo/query/acc.cgi?acc=GSE134303 Gene Expression Omnibus GSE134303.
