# Supplementary figures and images for: A microbiota-epigenetic circuit controls systematic circadian programs in the gut epithelium
Source: Front Syst Biol. 2023 Aug 8;3:1175306. doi: 10.3389/fsysb.2023.1175306 (PMC12312700; doi:10.3389/fsysb.2023.1175306)

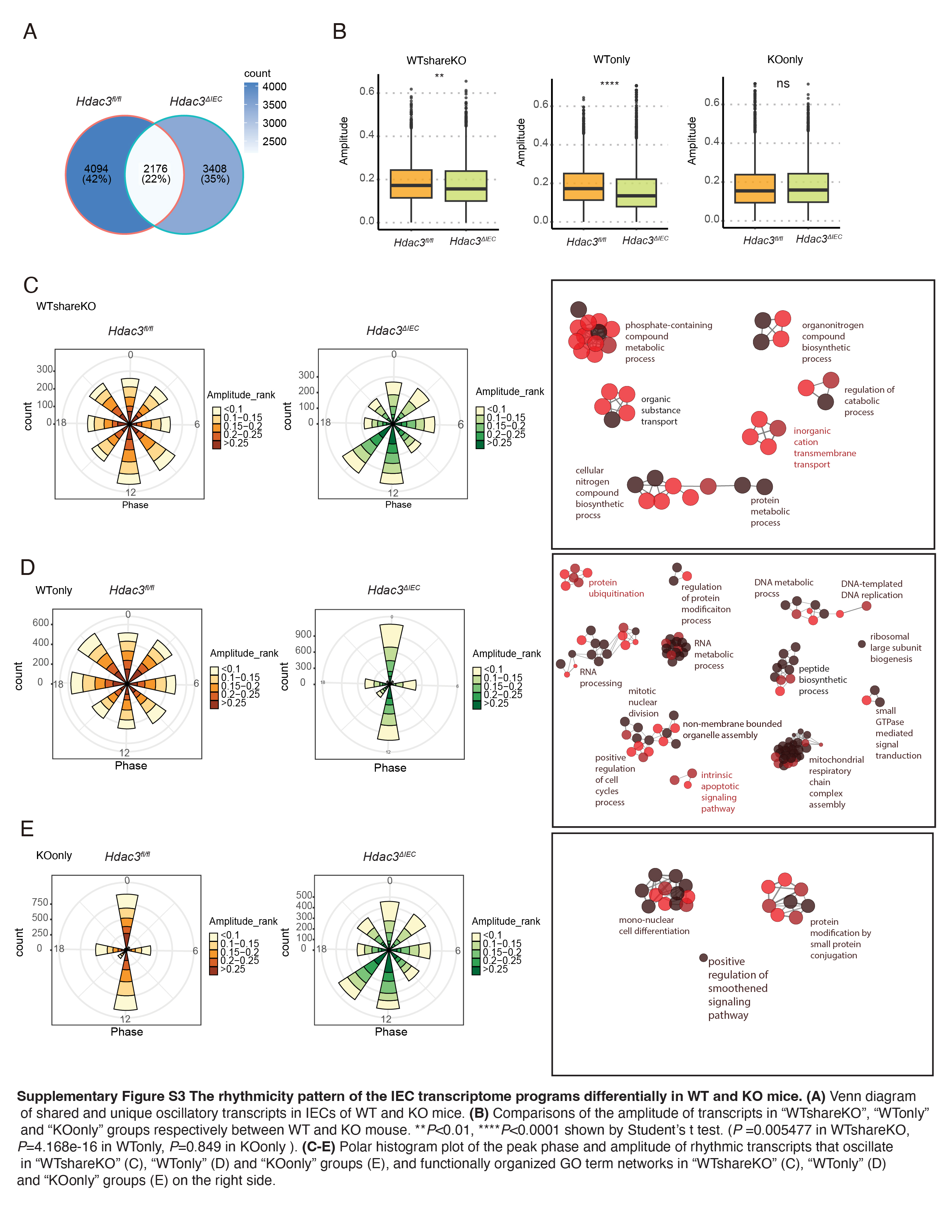

Supplement: Supplementary file 2 [file Image3.JPEG]

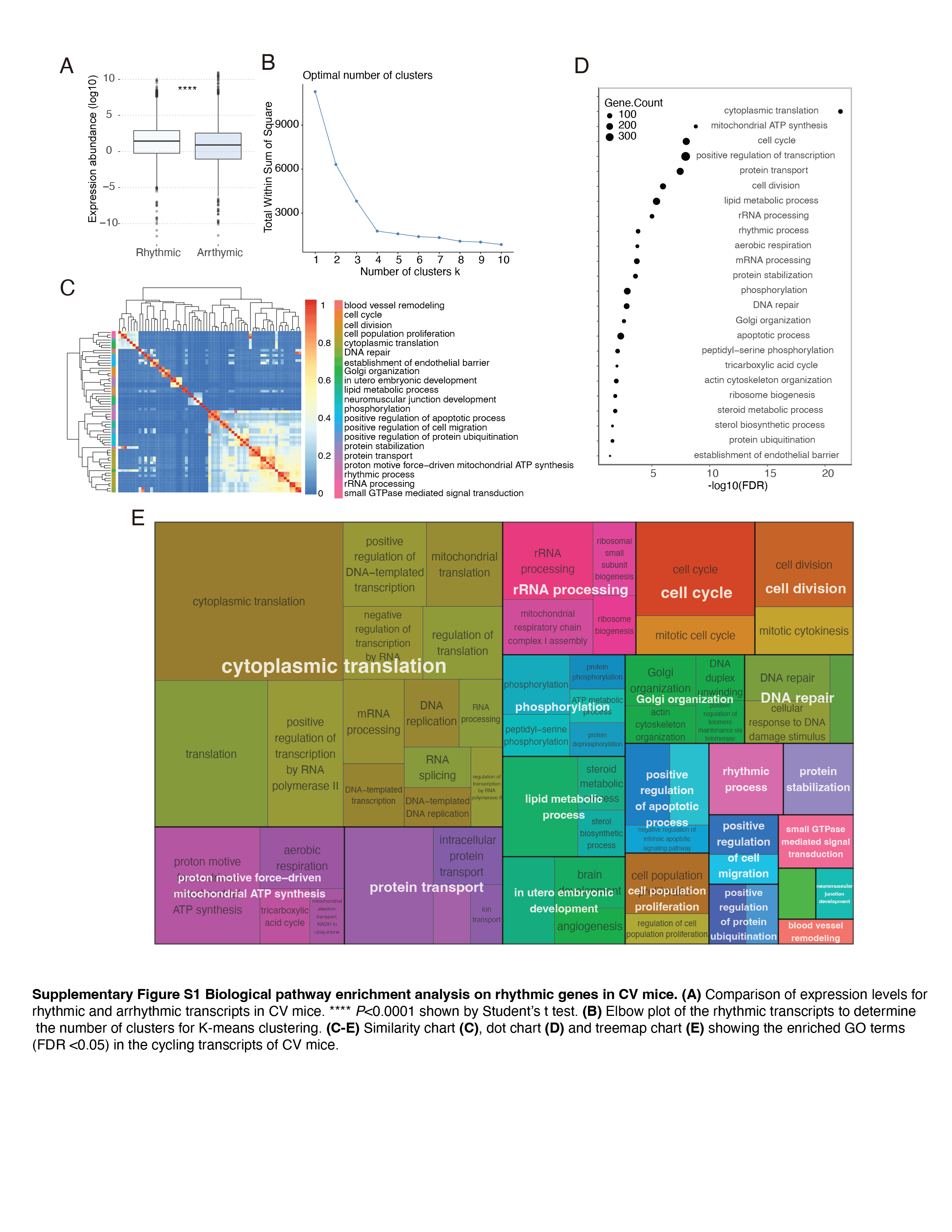

Supplement: Supplementary file 3 [file Image1.JPEG]

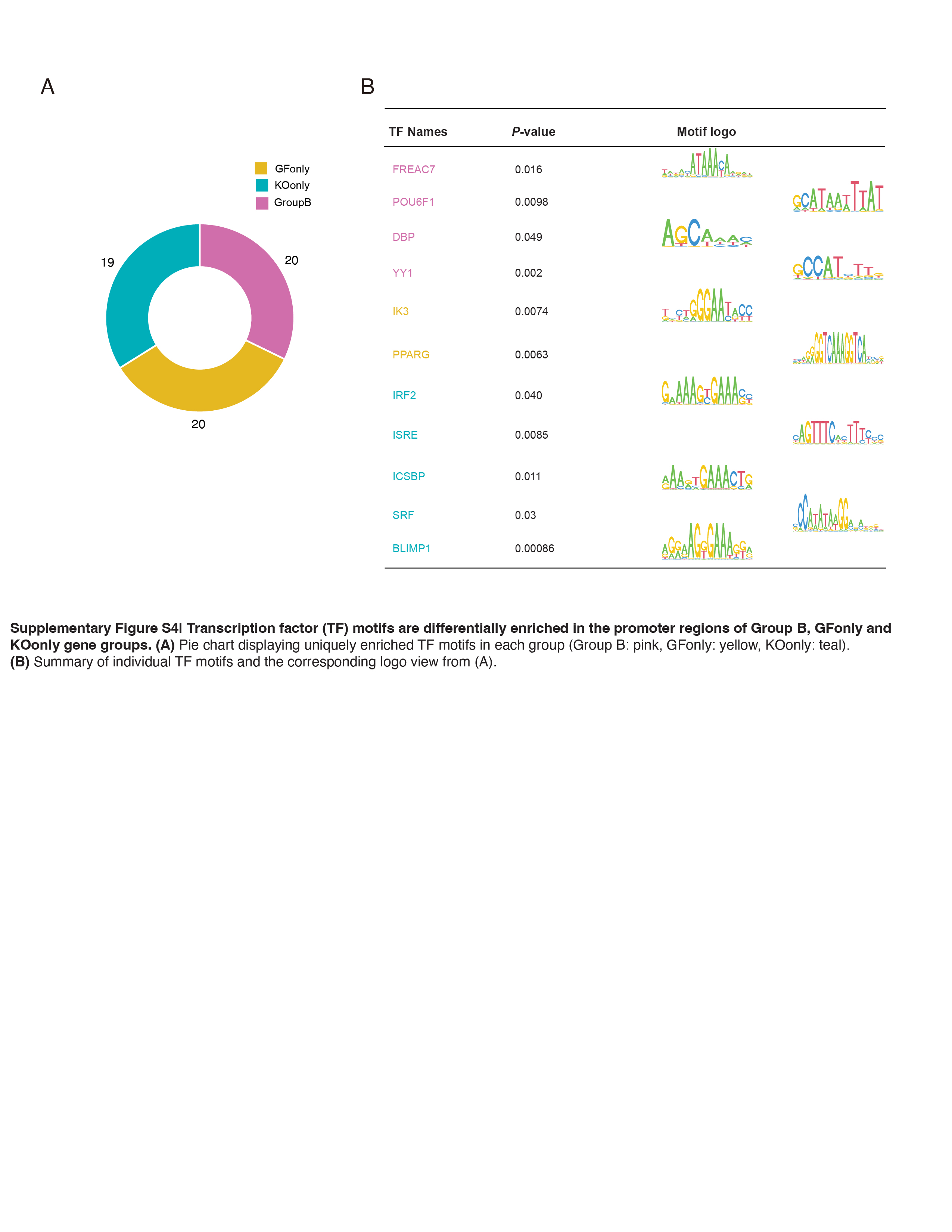

Supplement: Supplementary file 4 [file Image4.JPEG]

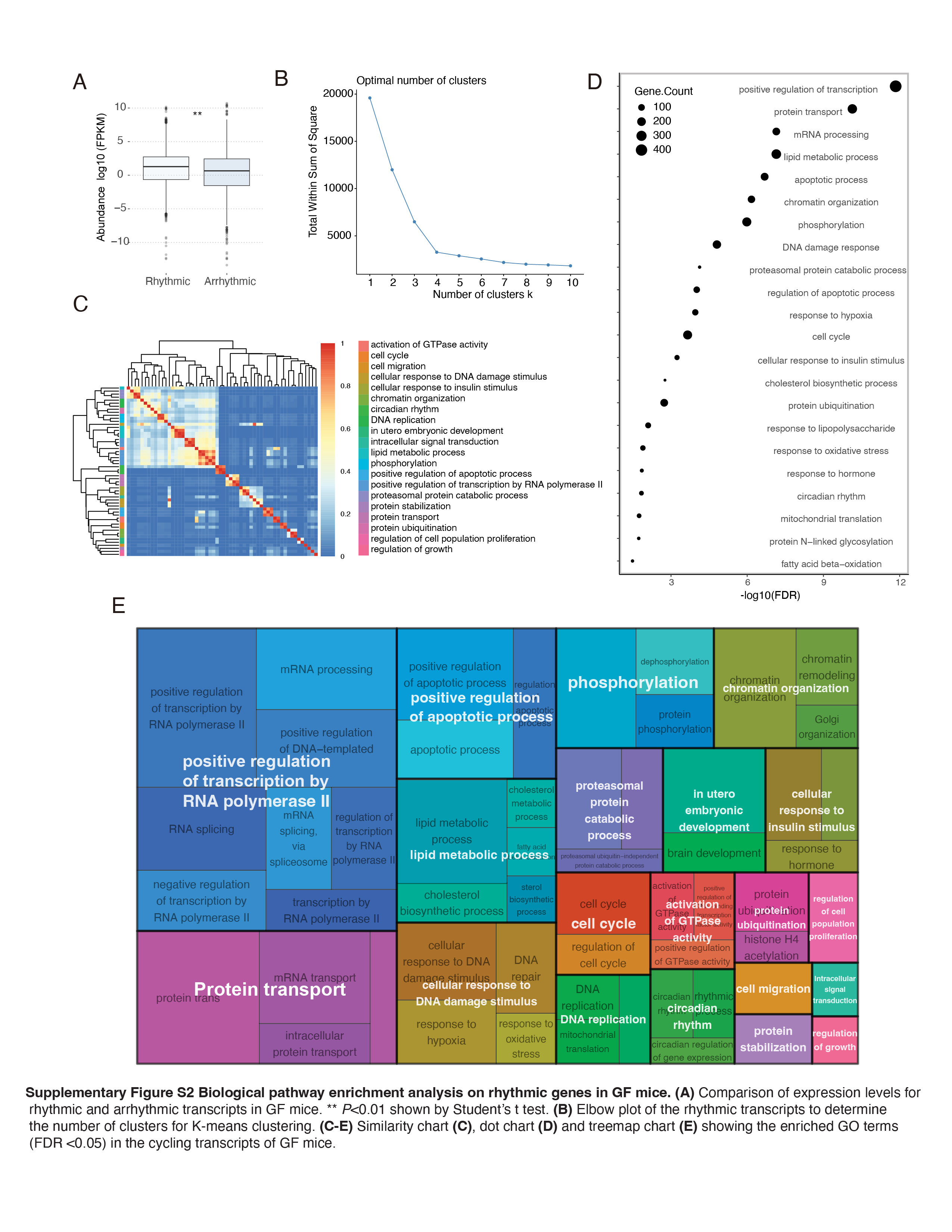

Supplement: Supplementary file 5 [file Image2.JPEG]
